# Thyroid function status and heterogeneity of efficacy of maintenance cognitive stimulation in late-life dementia: a stratified observational study of subclinical hypothyroidism/hyperthyroidism

**DOI:** 10.3389/fneur.2025.1710962

**Published:** 2026-01-12

**Authors:** Ruili Zhang, Yongling Zhou, Shan Wang, Feng Li, Xuan Zhao, Bowen Jia, Xiaxia Li, Junhui Yang, Ling Yang, Haiyuan Yu, Xiaomin Yu, Xuebing Xu

**Affiliations:** Ning An Hospital of Ningxia, Yinchuan, Ningxia Hui Autonomous Region, China

**Keywords:** cognitive stimulation therapy, dementia, observational cohort, subclinical hyperthyroidism, subclinical hypothyroidism, thyroid function, treatment-effect heterogeneity

## Abstract

**Background:**

Cognitive stimulation therapy (CST) and its maintenance phase (MCST) can benefit dementia. We evaluated treatment–effect heterogeneity by euthyroid versus subclinical hypothyroidism/hyperthyroidism during the 16-week maintenance period following CST.

**Methods:**

We conducted a prospective single-center cohort embedded in routine CST to MCST. All entrants to 7-week CST were assessed at baseline/8/16/24 weeks. Sixteen-week MCST occurred per usual care. Baseline TSH/FT4/FT3 are classified as euthyroid, subclinical hypothyroidism (SCH), or hyperthyroidism (SHyper). Co-primary outcomes were 24-week Montreal Cognitive Assessment (MoCA) and Zarit changes. We used doubly robust inverse-probability-of-treatment weighting combined with linear mixed-effects models to test MCST×thyroid interactions and controlled for multiple testing with a false-discovery-rate approach.

**Results:**

Of a total of 242 participants screened, 200 were enrolled, and 174 (87.0%) completed the 24-week study session. MCST continuation was 112 of 200 (56.0%) participants. Thyroid status was available for 196 participants, with 137 (69.9%) being euthyroid, 45 (23.0%) being SCH, and 14 (7.1%) being SHyper. The MCST×thyroid interaction for 24-week MoCA change was −0.9 (95% CI −1.6 to −0.2; *p* = 0.012; *q* = 0.012); MCST improved MoCA by +1.4 (95% CI +0.6 to +2.2) in euthyroid versus +0.5 (−0.4 to +1.3) in subclinical dysfunction. For Zarit, the interaction was +2.1 (95% CI +0.5 to +3.7; *p* = 0.011; *q* = 0.012), with larger burden reduction in euthyroid (−3.4; 95% CI −5.3 to −1.5) than subclinical dysfunction (−1.3; −3.1 to +0.4). Secondary outcomes favored MCST but were attenuated at higher TSH levels (spline *χ*^2^ = 8.9; *p* = 0.030). Agitation occurred in 3 of 200 participants (1.5%).

**Conclusion:**

MCST improved cognition and reduced caregiver burden over 24 weeks, with smaller benefits in subclinical thyroid dysfunction. Thyroid-aware personalization may better target maintenance cognitive interventions in late-life dementia.

## Introduction

Cognitive stimulation therapy (CST) and maintenance CST (MCST) are increasingly recognized as effective non-pharmacological interventions for dementia care. CST is a structured group-based intervention designed to enhance cognitive function and quality of life through activities that stimulate memory, executive function, and language skills (1, 2). Meta-analyses have demonstrated that CST has a small-to-moderate positive effect on cognitive function (3). The implementation of CST and MCST in real-world settings reveals variability in outcomes, which may be attributed to differences in intervention components, delivery methods, and participant characteristics (4). Furthermore, MCST, which extends the benefits of initial CST sessions, has been shown to improve quality of life and activities of daily living over longer periods, with additive effects when combined with medications such as acetylcholinesterase inhibitors (5, 6). While CST alone is cost-effective, the combined cost of CST and MCST may exceed thresholds for cost-effectiveness (7). Additionally, individual CST (iCST) has been explored as a means to enhance cognitive function and quality of life, though further research is needed to standardize protocols and confirm its efficacy (8). Overall, while CST and MCST offer promising benefits for dementia care, further research is needed to address the heterogeneity in treatment effects and optimize these interventions for diverse populations and settings (9, 10).

Subclinical thyroid dysfunction, particularly subclinical hypothyroidism, is prevalent among older adults and has been linked to cognitive impairment. A study in Mexico reported a 15.4% prevalence of subclinical hypothyroidism among older adults, correlating it with cognitive decline, especially in overt hypothyroidism cases (11). Conversely, a systematic review and meta-analysis indicated no significant association between subclinical hypothyroidism and cognitive decline in relatively healthy older adults, suggesting that, while cognitive impairment is common, it may not be directly influenced by subclinical thyroid dysfunction (12). In contrast, another meta-analysis focusing on subclinical hypothyroidism found that the risk of cognitive impairment and dementia was elevated only in individuals younger than 75 years and in those with higher TSH concentrations, with no significant association when all age groups were considered together, suggesting a biphasic, age-dependent pattern and potential misclassification when age-adjusted TSH ranges are not used (13). Additionally, researchers from India found a notable prevalence of hypothyroidism, with cognitive deficits observed in inadequately treated individuals (14). Furthermore, a study on older adults indicated that, while subclinical hyperthyroidism was associated with cognitive decline, subclinical hypothyroidism did not show a significant risk for dementia (15). Thus, while subclinical thyroid dysfunction is common, its impact on cognitive trajectories remains complex and requires further investigation.

Evidence on whether baseline thyroid status modifies cognitive stimulation therapy (CST) benefits during the post-CST maintenance phase remains limited. While some studies indicate that thyroid dysfunction, particularly subclinical hypothyroidism and hyperthyroidism, does not significantly impact cognitive function or dementia risk in older adults (16), other research studies highlights a potential sex-dependent relationship where higher thyroid-stimulating hormone (TSH) levels correlate with reduced odds of mild cognitive impairment in male individuals (17, 18). Additionally, a randomized controlled trial found no cognitive improvement from thyroxine replacement in elderly subjects with subclinical hypothyroidism (19). Thus, the interaction between thyroid status and cognitive outcomes in the context of maintenance CST requires further investigation to clarify these associations and their implications for treatment strategies.

This study aims to determine whether baseline thyroid status modifies the effectiveness of MCST on patient cognition and caregiver outcomes over approximately 24 weeks in real-world practice. By formally testing the MCST×thyroid interaction, we aim to quantify differences in both cognitive change and caregiver burden across thyroid-status strata and to generate clinically interpretable estimates of treatment–effect heterogeneity that can inform thyroid-aware implementation of MCST.

## Methods

### Study design and setting

This was a prospective, single-center, non-interventional cohort embedded in our hospital’s routine cognitive stimulation therapy (CST) service with an optional maintenance CST (MCST) phase from July 2022 to July 2024. All consecutive patients who entered a standard 7-week, group-based CST course were enrolled and followed at baseline and weeks 8, 16, and 24. The study complied with the Declaration of Helsinki and local regulations and received ethics committee approval before initiation. All participants or their legally authorized representatives provided written informed consent, with separate consent obtained from caregivers for their questionnaires.

### Participants and recruitment

Eligible participants were aged ≥60 years and had early-to-moderate dementia diagnosed by treating specialists in routine clinical practice. Dementia diagnoses and subtypes (Alzheimer’s disease, vascular dementia, mixed dementia, and other types) followed local/national guidelines and were based on informant history, neurological and mental-status examination, review of neuroimaging and laboratory work-up, and bedside cognitive testing. “Early-to-moderate” dementia referred to patients with clearly established cognitive decline and functional impact but preserved basic self-care and the ability to engage in group conversation and structured activities; we did not impose a fixed MoCA cut-off. At study baseline, the Montreal Cognitive Assessment (MoCA) was administered as the standardized global cognition measure. Eligible participants were also required to have sufficient sensory and communication abilities to participate in group sessions and an available informal caregiver willing to complete study questionnaires. The exclusion criteria were severe behavioral disturbance precluding group participation, prominent psychosis, or serious comorbid illness that, in the judgment of clinicians, made participation unsafe or impractical.

### CST and MCST program

CST was delivered in small, closed groups following the standardized CST manual adapted to our local context. Each session lasted approximately 60 min and was held twice-weekly for 7 weeks. Groups were led by trained clinicians (nurses/psychologists) who had completed CST training. At the start of each CST course, facilitators explained to participants and caregivers that the sessions were designed to support thinking and communication in an enjoyable way, not as a memory test, and described the schedule and group format in simple terms.

Each session followed a consistent structure: a brief orientation, a short warm-up activity, one or two theme-based cognitive activities (e.g., word games, categorization, reminiscence, and current events), and a short closing round. For each activity, facilitators provided short, clear verbal instructions, often demonstrated the task first, and used visual materials such as pictures, objects, and written words and gestures to support understanding. They encouraged responses from all group members, used open questions, and provided positive feedback rather than emphasizing correct/incorrect answers. When needed, instructions were repeated or broken into smaller steps, and prompts were adjusted to each participant’s abilities to maintain engagement.

Participants who continued according to usual care then received MCST, consisting of once-weekly sessions for an additional 16 weeks. CST sessions used the same group format and instructional style but focused on reviewing and reinforcing CST themes and introducing maintenance-oriented activities that built on previously learned material. The decision to continue was made jointly by clinicians, patients, and families without investigator influence. Session attendance was recorded for all CST and MCST sessions, and adherence was summarized as the proportion of sessions attended.

### Exposure ascertainment: baseline thyroid status

Before the first CST session, fasting morning blood samples were drawn to measure thyroid-stimulating hormone (TSH), free thyroxine (FT4), and free triiodothyronine (FT3) on the same platform. The adult reference intervals used by our hospital laboratory are TSH (0.27–4.20 mIU/L), FT4 (12–22 pmol/L), and FT3 (3.1–6.8 pmol/L), and these ranges are routinely applied to older adults in clinical practice. Antithyroid peroxidase (TPO) and antithyroglobulin (Tg) antibodies were obtained as part of routine work-up to aid interpretation but were not used to define thyroid categories or prespecified as effect modifiers. In the present analyses, we used these antibodies descriptively, reporting their baseline prevalence across thyroid strata as surrogate markers of autoimmune thyroid disease, but antibody-stratified outcome analyses were not performed because of limited sample size. Thyroid status was classified using laboratory reference ranges as euthyroid (TSH within reference, FT4/FT3 normal), subclinical hypothyroidism (SCH; TSH above reference with normal FT4/FT3), or subclinical hyperthyroidism (SHyper; TSH below reference with normal FT4/FT3), irrespective of prior clinical thyroid diagnosis or etiology (e.g., autoimmune or iatrogenic). Participants were not excluded on the basis of pre-existing thyroid disease or stable thyroid hormone or antithyroid drug therapy, provided that FT4 and FT3 were within reference, because we aimed to capture the functional thyroid milieu at CST entry in real-world practice. SHyper classification was based solely on this biochemical definition; we recorded thyroid-related medications but did not further subclassify SHyper into primary versus iatrogenic causes because of the small stratum size. We documented any thyroid-related medications and dose changes ordered by routine clinicians during follow-up and treated such changes as time-varying co-interventions in sensitivity analyses. The study team did not initiate or modify clinical management.

### Outcomes and covariates

Co-primary outcomes were change in global cognition from baseline to week 24, measured with the Montreal Cognitive Assessment (MoCA, 0–30), and change in caregiver burden over the same interval using the Zarit Burden Interview (ZBI). No additional research-mandated cognitive screening tool beyond the MoCA was required; other brief tests used at the clinician’s discretion in routine care were not consistently recorded and therefore were not analyzed in this cohort. Secondary outcomes included patient’s quality of life (Quality of Life in Alzheimer’s Disease, QoL-AD), behavioral and psychological symptoms (Neuropsychiatric Inventory-Questionnaire, NPI-Q), caregiver’s quality of life (EQ-5D-5L index), and caregiver’s anxiety and depression (GAD-7 and PHQ-9). Prespecified covariates were age, sex, education, dementia subtypes (Alzheimer’s disease, vascular dementia, mixed dementia, and other types), baseline MoCA, instrumental activities of daily living (IADL), comorbidity count, psychotropic medication use, calendar time of enrollment to account for secular trends, and session adherence. All instruments used validated Chinese versions available in the clinic, assessors were trained to standardized procedures, and outcomes were collected at baseline, week 8 (post-CST), week 16, and week 24.

### Sample size and power

The sample size of 200 was feasibility-driven by the service capacity and funding plan in the task book and was selected to provide adequate precision for mixed-effects estimation and treatment–effect heterogeneity (TEH) testing. Assuming approximately 10–15% attrition by week 24 and a prevalence of subclinical thyroid dysfunction of approximately 20–30%, simulations indicated >80% power to detect a moderate interaction effect on MoCA (a difference in MCST benefit between euthyroid and subclinical strata of ~0.8–1.0 points) at a two-sided *α* of 0.05 under repeated-measures mixed modeling with patient random intercepts. The primary modifier contrast was prespecified as any subclinical dysfunction versus euthyroid to ensure sufficient stratum counts, with three-level strata analyses considered exploratory.

### Statistical analysis

All analyses were two-sided and conducted in R (version 4.4). Since continuation to MCST was not randomized, we estimated the effect of MCST while reducing confounding using stabilized inverse-probability of treatment weights (IPTW) derived from logistic regression models that predicted the probability of continuing to MCST from baseline covariates (age, sex, education, subtype, baseline MoCA, IADL, comorbidity, psychotropic use, and calendar time) and program-related factors. We evaluated covariate balance using standardized mean differences and targeted ≤0.10 after weighting. To limit the undue influence of extreme weights, we stabilized IPTW by the marginal probability of MCST and truncated at the 1st and 99th percentiles before normalization to the sample size. We fit weighted linear mixed-effects models (participant random intercept) with fixed effects for time (categorical: baseline, 8, 16, and 24 weeks), MCST (yes/no), thyroid status, all lower-order terms, and the time×MCST×thyroid interaction. The week-24 MCST effect within each thyroid stratum and the between-strata interaction were obtained from this three-way interaction; robust standard errors were used. The primary estimand was the interaction contrast at week 24 comparing the MCST effect in euthyroid versus subclinical dysfunction. Three-level interactions (euthyroid, SCH, and SHyper) were assessed with Wald tests. For continuous thyroid function, we modeled TSH using restricted cubic splines with three degrees of freedom and tested a global MCST×spline interaction. Multiplicity for the two co-primary interaction tests (MoCA and ZBI) was controlled using the Benjamini–Hochberg false discovery rate (FDR) procedure targeting *q* < 0.05. Secondary interaction tests were reported with FDR-adjusted *q*-values across the secondary outcomes.

## Results

### Cohort assembly and follow-up

**Figure 1:**
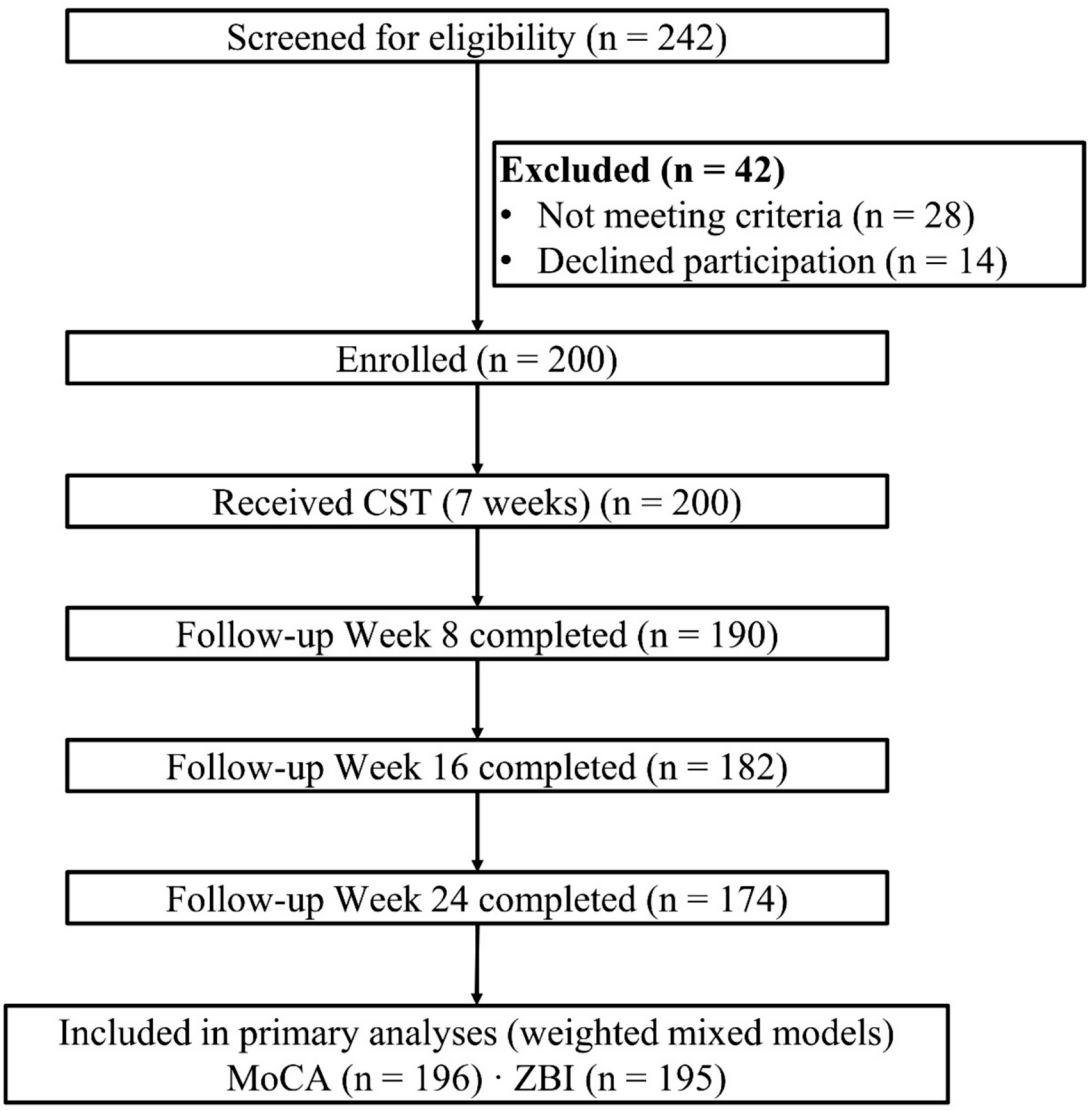
Flow diagram. A total of 242 individuals were screened, of which 42 were excluded (28 not meeting criteria, 14 declined). Two hundred were enrolled and received CST. Follow-up completion was 95.0% at week 8 (*n* = 190), 91.0% at week 16 (*n* = 182), and 87.0% at week 24 (*n* = 174). Included in primary analyses: MoCA (*n* = 196) and ZBI (*n* = 195).

A total of 242 individuals were screened, 42 were excluded, and 200 patients with early-to-moderate dementia were enrolled and initiated the 7-week CST program. Follow-up completion was 95.0% at week 8 (*n* = 190), 91.0% at week 16 (*n* = 182), and 87.0% at week 24 (*n* = 174), consistent with the prespecified project window and milestones. Thyroid classification was available for 196 participants (98.0%) (Figure 1).

### Baseline characteristics

At enrollment, groups defined by baseline thyroid status were broadly comparable (Table 1). Among the 196 participants with laboratories, 137 (69.9%) were euthyroid, 45 (23.0%) had SCH, and 14 (7.1%) had SHyper. The mean age of participants ranged from 76.8 to 78.3 years (*p* = 0.36), 57.1–60.0% were women (*p* = 0.97), and baseline cognition on the MoCA was 17.7–18.6 points (*p* = 0.24). Dementia subtype distribution, IADL, comorbidity count, and psychotropic use did not differ meaningfully across groups (all *p* > 0.40), and standardized mean differences comparing euthyroid with any subclinical dysfunction were small (≤0.23). Continuation to MCST occurred in 59.9, 53.3, and 50.0% of euthyroid, SCH, and SHyper participants, respectively, with no significant between-stratum differences (*p* = 0.56). We also report the baseline prevalence of positive TPO and Tg antibodies across thyroid strata as surrogate markers of autoimmune thyroid disease (Table 1).

**Table 1:** Baseline characteristics of the dementia cohort by thyroid status at enrollment.

Characteristics	Euthyroid (*n* = 137)	Subclinical hypothyroidism (SCH) (*n* = 45)	Subclinical hyperthyroidism (SHyper) (*n* = 14)	*p* (3-group)	SMD (Euth vs any subclinical)*
Age, years (mean ± SD)	77.0 ± 6.1	78.3 ± 6.5	76.8 ± 6.7	0.36^†^	0.15
Female, *n* (%)	80 (58.4%)	27 (60.0%)	8 (57.1%)	0.97^‡^	0.03
Education, years (median [IQR])	9 [6–12]	9 [6–12]	8 [6–11]	0.65^§^	—
Dementia subtype — AD, *n* (%)	85 (62.0)	25 (55.6)	8 (57.1)	0.77^‡^	—
Vascular, *n* (%)	30 (21.9%)	12 (26.7%)	2 (14.3%)		
Mixed, *n* (%)	14 (10.2%)	4 (8.9%)	2 (14.3%)		
Other, *n* (%)	8 (5.8%)	4 (8.9%)	2 (14.3%)		
Baseline MoCA (0–30), mean ± SD	18.6 ± 3.7	17.7 ± 3.9	18.0 ± 3.8	0.24^†^	0.23
IADL (0–8), mean ± SD	5.6 ± 1.3	5.3 ± 1.4	5.5 ± 1.2	0.41^†^	0.10
Comorbidity count, mean ± SD	2.1 ± 1.2	2.3 ± 1.3	2.0 ± 1.2	0.52^†^	0.16
Psychotropic use, *n* (%)	32 (23.4%)	13 (28.9%)	3 (21.4%)	0.74^‡^	0.11
Positive TPO antibody, *n* (%)	20 (14.6%)	22 (48.9%)	5 (35.7%)	<0.001	0.68
Positive Tg antibody, *n* (%)	15 (10.9%)	18 (40.0%)	3 (21.4%)	<0.001	0.54
Any thyroid autoantibody positive (TPO or Tg), *n* (%)	25 (18.2%)	27 (60.0%)	6 (42.9%)	<0.001	0.77
Continued to MCST, *n* (%)	82 (59.9%)	24 (53.3%)	7 (50.0%)	0.56^‡^	0.11

^*^SMD compares Euthyroid vs. Subclinical (SCH+SHyper). ^†^ANOVA; ^‡^*χ*^2^ test; ^§^Kruskal–Wallis. SCH, subclinical hypothyroidism; SHyper, subclinical hyperthyroidism; MoCA, Montreal Cognitive Assessment; IADL, Instrumental Activities of Daily Living; MCST, Maintenance Cognitive Stimulation Therapy; SMD, standardized mean difference.

### Primary outcome: global cognition

**Figure 2:**
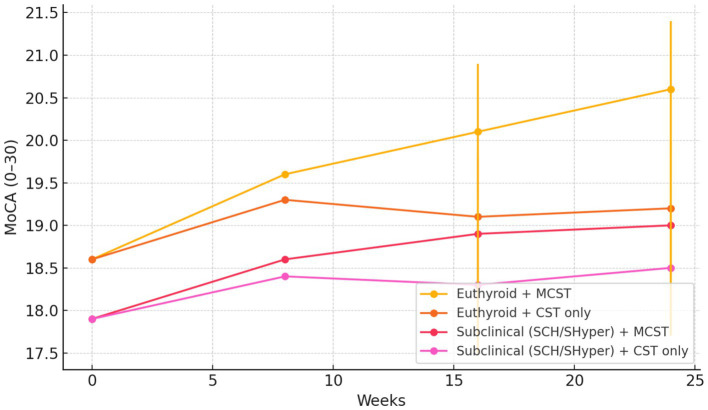
Model-estimated cognitive trajectories (MoCA) by thyroid status and MCST exposure. Predicted means from the doubly robust mixed model with IPTW show greater separation of MCST versus CST in the euthyroid stratum than in subclinical dysfunction (SCH/SHyper). Error bars at weeks 16 and 24 denote 95% CIs for plotted means. The MCST × Subclinical interaction at 24 weeks is −0.9 MoCA points (95% CI −1.6 to −0.2; *p* = 0.012; *q*_FDR_ = 0.012), indicating attenuated MCST benefit with subclinical thyroid dysfunction.

In the weighted, doubly robust mixed-effects model, the covariate balance between continuation groups improved significantly, with the mean standardized mean difference decreasing from 0.18 (maximum 0.31) pre-weighting to 0.04 (maximum 0.09) post-weighting, indicating adequate balance. The MCST×thyroid interaction at week 24 was significant for MoCA change (interaction contrast −0.9 points; 95% CI −1.6 to −0.2; *p* = 0.012; FDR *q* = 0.012), indicating that the cognitive benefit of MCST differed by thyroid status (Table 2). Among euthyroid participants, MCST was associated with a small-to-moderate improvement in global cognition compared with CST alone (adjusted MCST vs. CST difference +1.4 points; 95% CI +0.6 to +2.2; *p* = 0.001). Among participants with subclinical thyroid dysfunction (SCH + SHyper), the estimated MCST advantage was smaller and not statistically significant (+0.5; 95% CI −0.4 to +1.3; *p* = 0.28) (Table 2). Thus, MCST remained directionally beneficial across thyroid strata but produced attenuated cognitive gains in subclinical dysfunction compared with euthyroid status. A global three-level interaction across euthyroid, SCH, and SHyper was also supported (Wald *F* = 3.5; *p* = 0.032). However, the subgroup-specific estimates for SHyper are based on only 14 participants and are therefore highly imprecise, so we treat these as exploratory and refrain from drawing firm conclusions for SHyper. Model-estimated trajectories illustrated greater separation between MCST and CST curves in the euthyroid stratum over 24 weeks (Figure 2).

**Table 2:** Primary outcome: global cognition (MoCA) adjusted 24-week effects and interaction.

Analysis	Group/Contrast	Adjusted 24-week change from baseline, mean (95% CI)	MCST vs CST difference at 24 wk (95% CI)	*p* (difference)	*p*_interaction	*q*_FDR (co-primary)
Euthyroid	MCST	+2.0 (+1.2 to +2.8)				
	CST-only	+0.6 (−0.2 to +1.4)	+1.4 (+0.6 to +2.2)	0.001		
Subclinical dysfunction (SCH+SHyper)	MCST	+1.1 (+0.1 to +2.1)				
	CST-only	+0.6 (−0.2 to +1.4)	+0.5 (−0.4 to +1.3)	0.28		
Interaction (TEH)	MCST×Subclinical vs. MCST×Euthyroid	—	−0.9 (−1.6 to −0.2)	0.012	0.012	0.012

Model: Doubly robust linear mixed-effects (patient random intercept), stabilized IPTW for MCST continuation; covariates: age, sex, education, subtype, baseline MoCA, IADL, comorbidity, psychotropic use, calendar time, and session adherence. Robust SEs. Global 3-level interaction (Euth vs. SCH vs. SHyper): Wald *F* = 3.5, *p* = 0.032. FDR (*q*) uses Benjamini–Hochberg across the two co-primary interaction tests.

### Co-primary outcome: caregiver burden

For caregiver burden, the MCST × thyroid interaction at week 24 was similarly significant (difference-of-differences in ZBI + 2.1 points; 95% CI +0.5 to +3.7; *p* = 0.011; FDR *q* = 0.012), again indicating that the magnitude of benefit depended on thyroid status (Table 3). In the euthyroid group, MCST reduced ZBI by −3.4 points compared with CST alone (95% CI −5.3 to −1.5; *p* = 0.001), whereas in the subclinical dysfunction (SCH + SHyper) group, the estimated reduction was −1.3 points (95% CI −3.1 to +0.4; *p* = 0.14). This pattern mirrors the MoCA results: caregivers of euthyroid participants experienced larger and more certain burden reductions, while caregivers of participants with subclinical hypothyroidism or hyperthyroidism experienced smaller, less certain gains.

**Table 3:** Primary outcome: caregiver burden (Zarit) adjusted 24-week effects and interaction.

Analysis	Group/Contrast	Adjusted 24-week change from baseline, mean (95% CI)	MCST vs. CST difference at 24 weeks (95% CI)	*p* (difference)	*p*_interaction	*q*_FDR (co-primary)
Euthyroid	MCST	−4.9 (−6.6 to −3.2)				
	CST-only	−1.5 (−3.0 to 0.0)	−3.4 (−5.3 to −1.5)	0.001		
Subclinical dysfunction (SCH+SHyper)	MCST	−2.8 (−5.1 to −0.5)				
	CST-only	−1.5 (−3.5 to +0.5)	−1.3 (−3.1 to +0.4)	0.14		
Interaction (TEH)	MCST×Subclinical vs. MCST×Euthyroid	—	+2.1 (+0.5 to +3.7)*	0.011	0.011	0.012

*Positive value denotes a smaller MCST-related reduction in ZBI among subclinical dysfunction vs. euthyroid. Model, weighting, and FDR as in Table 2.

### Secondary outcomes

Patterns were directionally consistent across secondary domains (Table 4). MCST improved patient’s quality of life more in euthyroid than in subclinical dysfunction (QoL-AD interaction −1.9; 95% CI −3.6 to −0.2; *p* = 0.028; FDR *q* = 0.082) and reduced behavioral symptoms to a greater extent in euthyroid participants (NPI-Q interaction +1.4; 95% CI +0.1 to +2.7; *p* = 0.041; FDR *q* = 0.082). Interactions for caregiver QoL and anxiety were not statistically significant after multiplicity control (*p* = 0.18 and *p* = 0.22, respectively). Modeling TSH as a continuous modifier using restricted cubic splines yielded a significant global MCST×TSH interaction (*χ*^2^ = 8.9; *p* = 0.030), indicating that MCST benefits varied across the TSH continuum and were particularly attenuated at higher, subclinical-hypothyroid-range TSH values, whereas confidence intervals at the lowest TSH values (corresponding to the small SHyper subgroup) were wide and compatible with a range of possible effects, limiting firm inference in that region of the TSH spectrum.

**Table 4:** Secondary outcomes and sensitivity analyses: MCST and CST effects at 24 weeks by thyroid status.

Outcome/Analysis	Euthyroid: MCST vs. CST effect at 24 weeks (95% CI)	Subclinical: MCST vs. CST effect (95% CI)	Interaction (Subclinical vs Euth) (95% CI)	*p*_interaction	*q*_FDR (secondary)**
QoL-AD (patient)	+3.6 (+2.0 to +5.1)	+1.7 (−0.2 to +3.6)	−1.9 (−3.6 to −0.2)	0.028	0.082
NPI-Q (behavioral symptoms)	−2.1 (−3.4 to −0.9)	−0.7 (−2.4 to +0.9)	+1.4 (+0.1 to +2.7)	0.041	0.082
Caregiver QoL (EQ-5D index)	+0.05 (+0.02 to +0.08)	+0.02 (−0.02 to +0.06)	−0.03 (−0.07 to +0.01)	0.18	0.22
Caregiver anxiety (GAD-7)	−1.3 (−2.1 to −0.5)	−0.6 (−1.8 to +0.6)	+0.7 (−0.5 to +1.9)	0.22	0.22
Continuous TSH×MCST (restricted cubic spline, 3 degrees of freedom)	—	—	Global spline interaction: *χ*^2^ = 8.9	0.030	—
Covariate balance (IPTW)	Pre-weighting mean SMD = 0.18 (max 0.31)	Post-weighting mean SMD = 0.04 (max 0.09)	—	—	—
Per-protocol (≥70% sessions)	+1.6 (+0.6 to +2.6)	+0.6 (−0.5 to +1.6)	−1.0 (−1.9 to −0.1)	0.028	—
Safety	No study-related serious adverse events; transient agitation 3/200 (1.5%) during sessions	—	—	—	—

^**^FDR q-values computed across the four secondary interactions (QoL-AD, NPI-Q, caregiver QoL, and GAD-7).

### Sensitivity and robustness

The findings were robust in multiple analyses (Table 4). Per-protocol analyses restricted to participants attending ≥70% of sessions demonstrated a greater cognitive benefit of MCST in euthyroid patients and preserved the interaction effect (−1.0 MoCA points; 95% CI −1.9 to −0.1; *p* = 0.028). Complete case analyses yielded estimates similar in direction and magnitude to those from the primary multiply imputed models, and conclusions were unchanged when alternative outcome distributions were specified for skewed measures. When we restricted the subclinical group to SCH only (excluding SHyper), the pattern of effect modification at 24 weeks remained broadly similar (Supplementary Table S1). Excluding observations with extreme weights or large residuals did not materially alter the interaction estimates.

No study-related serious adverse events occurred. Three participants (1.5%) experienced transient agitation during group sessions that resolved with brief reassurance and did not necessitate clinical intervention (Table 4).

## Discussion

In this prospective, single-center cohort embedded in our routine CST program with an optional MCST phase, we followed consecutively enrolled patients with early-to-moderate dementia and classified their thyroid status at baseline using TSH, FT4, and FT3. Over 24 weeks, MCST was associated with small-to-moderate improvements in global cognition and caregiver burden. MCST-related improvements in patients’ quality of life and reductions in behavioral symptoms were larger in euthyroid patients, whereas the effects were smaller and estimated with less precision in the subclinical thyroid dysfunction group. Our exploratory three-level analyses suggested that both SCH and SHyper may contribute to this pattern, but estimates for SHyper were based on only 14 participants and were therefore imprecise. Consistent with this finding, the continuous TSH analysis showed more robust attenuation of MCST benefits at higher TSH values within the subclinical-hypothyroid range, whereas the spline confidence bands remained wide at very low TSH, indicating limited information about the SHyper range. Taken together, these findings indicate that, while MCST remains beneficial overall in late-life dementia, the magnitude of benefit differs by thyroid status, demonstrating treatment–effect heterogeneity rather than an absence of effect in any subgroup.

In this real-world cohort of older adults with early-to-moderate dementia, we found that baseline thyroid status clearly modified the 24-week effectiveness of MCST on both cognition and caregiver burden. After inverse-probability weighting and adjustment, MCST was associated with a small-to-moderate cognitive benefit in euthyroid participants (adjusted MCST vs. CST difference of about +1.4 MoCA points) and a clinically relevant reduction in caregiver burden (−3.4 ZBI points). In contrast, participants with subclinical thyroid dysfunction (subclinical hypothyroidism or subclinical hyperthyroidism) showed only a modest and statistically uncertain cognitive gain (+0.5 MoCA points) and a smaller, non-significant reduction in caregiver burden (−1.3 ZBI points). The pre-specified two-level interaction (euthyroid vs. any subclinical dysfunction) and the exploratory three-level test (euthyroid vs. subclinical hypothyroidism vs. subclinical hyperthyroidism) were both statistically significant, but the subclinical hyperthyroidism estimates were imprecise because of the small subgroup (*n* = 14). Modeling TSH as a continuous modifier with restricted cubic splines yielded a significant global MCST×TSH interaction, with attenuation of MCST benefits most clearly supported at higher TSH values, where data density was greatest, and wide confidence intervals at very low TSH, where few participants had subclinical hyperthyroidism. These effect sizes and patterns are consistent with small-to-moderate benefits of CST/MCST reported in randomized trials and meta-analyses in mild-to-moderate dementia and Alzheimer’s disease, suggesting that MCST remains beneficial overall, although the magnitude of benefit is reduced in the presence of subclinical thyroid dysfunction (2, 3, 5, 9, 20).

Our findings also fit within a complex epidemiologic literature on subclinical thyroid dysfunction and late-life cognition. Trials and reviews of groups CST and MCST in dementia consistently show modest improvement in global cognition and quality of life across Alzheimer’s disease, vascular dementia, and mixed dementia, with similar benefits in a culturally adapted program for Chinese people with dementia (2, 3, 5, 9, 20, 21). In contrast, community-based studies of thyroid function in older adults without dementia have yielded mixed results: observational cohorts in Mexico and India suggest that hypothyroidism and subclinical hypothyroidism are common and may be associated with poorer cognitive performance, especially when inadequately treated, whereas a systematic review and an individual-participant-data meta-analysis did not find a consistent association between subclinical hypothyroidism and global cognitive function in people aged more than 60 (11, 12, 14, 16). Prospective cohorts and meta-analyses indicate that subclinical hyperthyroidism may increase the risk of dementia, while subclinical hypothyroidism often shows no clear association with dementia or accelerated MMSE decline (15, 22). The Rotterdam Study reported that higher and high-normal thyroid function is associated with an increased risk of dementia and Alzheimer’s disease, independent of overt vascular brain lesions, and an age-stratified meta-analysis suggests that subclinical hypothyroidism confers an increased risk of cognitive impairment and dementia only in individuals younger than 75 years with higher TSH, but not in the oldest old (13, 23). A trial of thyroxine replacement in community-dwelling older adults with subclinical hypothyroidism showed no improvement in cognitive function (19). Together, these studies describe a heterogeneous and partly age-dependent relationship between subclinical thyroid states and dementia risk. They focus on incident dementia in community-based samples rather than treatment response in established dementia; therefore, they do not directly predict our thyroid-stratified MCST effects but provide important background for interpreting them.

Several neurobiological and clinical mechanisms may plausibly contribute to the smaller MCST benefits observed in dementia patients with subclinical thyroid dysfunction, although these mechanisms were not directly measured in our cohort and should therefore be regarded as hypothetical. Thyroid hormones are crucial for synaptogenesis, myelination, and hippocampal development and continue to modulate synaptic plasticity and adult hippocampal neurogenesis, processes that underpin learning and memory in Alzheimer’s disease and other dementia types (24, 25). It is therefore plausible that higher TSH and reduced thyroid hormone signaling in subclinical hypothyroidism could blunt learning-related plasticity and slow consolidation, diminishing the cognitive gains achievable with repeated group stimulation over 24 weeks. In addition, thyroid–brain interaction studies show that hypothyroid states, including subclinical hypothyroidism, are associated with depressive symptoms, fatigue, psychomotor slowing, and attentional difficulties, and meta-analytic evidence supports an association between subclinical hypothyroidism and depression (26, 27). In people with dementia, this symptom profile may lower engagement and persistence in MCST sessions, contributing to smaller observed improvements. For subclinical hyperthyroidism, pooled cohort analyses indicate increased risks of coronary heart disease, atrial fibrillation, and cardiovascular mortality, and imaging studies from the Rotterdam cohort suggests that altered thyroid status is associated with changes in brain circulation and cerebral perfusion (28, 29). These cardiovascular and perfusion changes could, in principle, reduce cognitive reserve and responsiveness to cognitive stimulation in dementia and Alzheimer’s disease, especially over longer maintenance phases. However, we did not collect systematic cardiovascular or perfusion measures, and the subclinical hyperthyroidism group was small, so these mechanisms remain speculative in the context of our data and should be framed as hypotheses that are consistent with, but not proven by, the observed continuous TSH interaction.

Clinically, our findings argue for “thyroid-aware” rather than “thyroid-selective” use of MCST in dementia. Current dementia guidelines already recommend group CST as a core non-pharmacological intervention for people with mild-to-moderate dementia, and person-centered psychosocial programs have been shown to improve quality of life and reduce agitation in nursing home residents with dementia (4, 30). Our data support continuing CST and MCST as standard care for dementia and Alzheimer’s disease, while using routinely available thyroid tests (TSH, FT4 ± FT3) help set realistic expectations and tailor support. For euthyroid patients, small-to-moderate gains in cognition and caregiver burden over 24 weeks are likely and can be communicated to families. For patients with subclinical hypothyroidism or subclinical hyperthyroidism, MCST should still be offered, but teams can anticipate smaller and less certain gains, pay closer attention to fatigue, mood, and attentional problems during sessions, adjust pacing and breaks in subclinical hypothyroidism, and ensure appropriate cardiovascular risk assessment and fall-risk management in subclinical hyperthyroidism, in line with existing thyroid guidelines (31, 32). At the same time, randomized trials and meta-analyses in older adults with subclinical hypothyroidism show that levothyroxine therapy does not meaningfully improve cognition, quality of life, or thyroid-related symptoms, indicating that simply normalizing TSH pharmacologically cannot currently be recommended as a strategy to enhance MCST response in dementia (19, 33, 34). Instead, thyroid-aware personalization should focus on assessment, expectation management, and adjustment of psychosocial care within established CST/MCST and cultural-adaptation frameworks (21, 35).

Important limitations of this study should be acknowledged. The single-center, observational design and clinician−/family-driven continuation to MCST limit generalizability and leave residual confounding possible despite careful adjustment. Thyroid status was classified from a single baseline panel using standard adult reference ranges rather than age-adjusted intervals, which may have misclassified some very old participants with modest TSH elevations. The relatively high prevalence but small size of the subclinical hyperthyroidism group, which was not further classified into primary versus iatrogenic causes, together with our inability to stratify by autoimmune status despite measuring thyroid antibodies, limits etiologic interpretation. The subclinical hyperthyroidism subgroup was small (*n* = 14), so subgroup-specific estimates for SHyper are highly uncertain and contributed to wide confidence intervals at low TSH values in the spline analyses. As a result, we could not draw firm conclusions about MCST effectiveness in SHyper, and we consider those estimates exploratory. We also lacked systematic measures of cardiovascular status, arrhythmias, or cerebral perfusion, so any proposed vascular or autonomic mechanisms remain speculative. Dementia diagnoses and staging were based on routine clinical assessment rather than research-grade criteria. “Early-to-moderate” severity was defined clinically rather than via a strict MoCA cutoff, and the MoCA was the only cognitive test collected consistently, precluding more granular domain-specific analyses. Finally, although attrition was modest and handled with mixed-effects models, the assumption that missing data are conditionally random may not fully hold, and both patient- and caregiver-reported scales are vulnerable to measurement error and reporting bias. Taken together, these considerations mean that our findings should be viewed as exploratory and hypothesis-generating, pointing toward future larger, multi-center studies or pragmatic trials that deliberately enroll sufficient numbers of patients with subclinical hypothyroidism and subclinical hyperthyroidism, incorporate repeated thyroid measurements with age-adjusted reference ranges, distinguish primary from iatrogenic dysfunction and consider autoimmune status as a separate modifier, and embed mechanistic work to clarify pathways and test thyroid-aware tailoring of CST content and pacing in stratified or personalized intervention protocols.

In summary, in this routine-care cohort of older adults with early-to-moderate dementia, MCST improved global cognition and reduced caregiver burden over 24 weeks, and baseline thyroid status emerged as an accessible biological factor that helps explain how much benefit each person receives. MCST should therefore continue to be offered to all eligible patients with dementia, while thyroid-aware personalization is used to guide expectations, adjust pacing and support, and inform wider multidisciplinary care rather than to restrict access to treatment. These findings now warrant replication and refinement in larger, multi-center cohorts with richer mechanistic measures, with the ultimate goal of enabling more individualized, biologically informed non-pharmacological care for people living with dementia.

## Data Availability

The raw data supporting the conclusions of this article will be made available by the authors, without undue reservation.
