# Supplementary material for: Thyroid function status and heterogeneity of efficacy of maintenance cognitive stimulation in late-life dementia: a stratified observational study of subclinical hypothyroidism/hyperthyroidism
Source: Front Neurol. 2026 Jan 12;16:1710962. doi: 10.3389/fneur.2025.1710962 (PMC12832453; doi:10.3389/fneur.2025.1710962)
Supplement: Supplementary file 1 [file Table_1.docx]

**TABLE S1. Sensitivity analysis restricting subclinical thyroid dysfunction to subclinical hypothyroidism (SCH only): adjusted 24‑week MCST effects and MCST×thyroid interactions for primary outcomes**

**Panel A. Global cognition (MoCA)**

| **Analysis** | **Group/Contrast** | **Adjusted 24‑week change from baseline, mean (95% CI)** | **MCST vs CST difference at 24 wk (95% CI)** | **p (difference)** | **p_interaction** |
| --- | --- | --- | --- | --- | --- |
| **Euthyroid** | MCST | +2.0 (+1.2 to +2.8) |  |  |  |
|  | CST only | +0.6 (−0.2 to +1.4) | **+1.4 (+0.6 to +2.2)** | 0.001 | — |
| **Subclinical hypothyroidism (SCH only)** | MCST | +1.3 (+0.2 to +2.4) |  |  |  |
|  | CST only | +0.7 (−0.3 to +1.7) | **+0.6 (−0.5 to +1.7)** | 0.29 | — |
| **Interaction (TEH)** | MCST×SCH vs MCST×Euthyroid | — | **−0.8 (−1.7 to +0.1)** | — | 0.08 |

**Panel B. Caregiver burden (Zarit Burden Interview, ZBI)**

| **Analysis** | **Group/Contrast** | **Adjusted 24‑week change from baseline, mean (95% CI)** | **MCST vs CST difference at 24 wk (95% CI)** | **p (difference)** | **p_interaction** |
| --- | --- | --- | --- | --- | --- |
| **Euthyroid** | MCST | −4.9 (−6.6 to −3.2) |  |  |  |
|  | CST only | −1.5 (−3.0 to 0.0) | **−3.4 (−5.3 to −1.5)** | 0.001 | — |
| **Subclinical hypothyroidism (SCH only)** | MCST | −3.0 (−5.5 to −0.5) |  |  |  |
|  | CST only | −1.6 (−3.6 to +0.4) | **−1.4 (−3.3 to +0.5)** | 0.15 | — |
| **Interaction (TEH)** | MCST×SCH vs MCST×Euthyroid | — | **+2.0 (−0.1 to +4.1)** | — | 0.06 |
